# Segmentation-Guided Hybrid Deep Learning for Pulmonary Nodule Detection and Risk Prediction from Multi-Cohort CT Images

**DOI:** 10.3390/diseases14010021

**Published:** 2026-01-06

**Authors:** Gomavarapu Krishna Subramanyam, Kundojjala Srinivas, Veera Venkata Raghunath Indugu, Dedeepya Sai Gondi, Sai Krishna Gaduputi Subbammagari

**Affiliations:** 1 Department of Computer Science and Engineering, Koneru Lakshmaiah Education Foundation, Hyderabad 500075, Telangana, India; gkrishnasubramanyam03@gmail.com; 2 Department of Computer Science and Engineering, Keshav Memorial Institute of Technology, Narayanaguda, Hyderabad 500029, Telangana, India; 3 Independent Researcher, Exton, PA 19341, USA; 4 Independent Researcher, Dallas, TX 75001, USA; 5 Department of Computer Science, University of Nebraska Lincoln, 7506 Poppleton Plaza, Omaha, NE 68124, USA

**Keywords:** lung cancer, LDCT, pulmonary nodule detection, malignancy classification, DenseNet, Swin Transformer

## Abstract

Background: Lung cancer screening using low-dose computed tomography (LDCT) demands not only early pulmonary nodule detection but also accurate estimation of malignancy risk. This remains challenging due to subtle nodule appearances, the large number of CT slices per scan, and variability in radiological interpretation. The objective of this study is to develop a unified computer-aided detection and diagnosis framework that improves both nodule localization and malignancy assessment while maintaining clinical reliability. Methods: We propose Seg-CADe-CADx, a dual-stage deep learning framework that integrates segmentation-guided detection and malignancy classification. In the first stage, a segmentation-guided detector with a lightweight 2.5D refinement head is employed to enhance nodule localization accuracy, particularly for small nodules with diameters of 6 mm or less. In the second stage, a hybrid 3D DenseNet–Swin Transformer classifier is used for malignancy prediction, incorporating probability calibration to improve the reliability of risk estimates. Results: The proposed framework was evaluated on established public benchmarks. On the LUNA16 dataset, the system achieved a competitive performance metric (CPM) of 0.944 for nodule detection. On the LIDC-IDRI dataset, the malignancy classification module achieved a ROC-AUC of 0.988, a PR-AUC of 0.947, and a specificity of 97.8% at 95% sensitivity. Calibration analysis further demonstrated strong agreement between predicted probabilities and true malignancy likelihoods, with an expected calibration error of 0.209 and a Brier score of 0.083. Conclusions: The results demonstrate that hybrid segmentation-guided CNN–Transformer architectures can effectively improve both diagnostic accuracy and clinical reliability in lung cancer screening. By combining precise nodule localization with calibrated malignancy risk estimation, the proposed framework offers a promising tool for supporting radiologists in LDCT-based lung cancer assessment.

## 1. Introduction

Lung cancer is the foremost cause of cancer-related mortality globally, with approximately 2.2 million new cases and 1.8 million deaths recorded in 2020 [1]. Survival outcomes are closely associated with the stage at which the disease is identified, as early-stage detection can substantially improve the 5-year survival rate, often exceeding 60–70%, while late-stage diagnoses result in survival rates below 10%. Large-scale randomized trials such as NLST [2] and NELSON [3] have confirmed the effectiveness of low-dose computed tomography (LDCT) screening, reporting significant reductions in lung cancer mortality compared to conventional imaging techniques.

**Figure 1:**
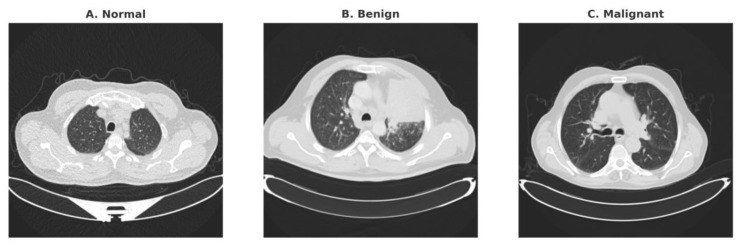
Representative LDCT slices from the dataset used in this study: (**A**) Normal lung parenchyma, (**B**) Benign-appearing pulmonary nodule, and (**C**) Malignant pulmonary nodule.

Despite these clinical advantages, widespread adoption of LDCT screening introduces several practical challenges. Radiologists must interpret thousands of image slices, where pulmonary nodules frequently present as small, subsolid, or vessel-attached lesions that are difficult to distinguish from surrounding anatomical structures. Such subtle manifestations lead to variability in reader interpretation, elevated false-positive rates, and unnecessary follow-up examinations, thereby increasing both healthcare costs and patient anxiety [4,5]. Figure 1 provides representative LDCT slices, including normal lung parenchyma, a benign pulmonary nodule, and a malignant pulmonary nodule.

To alleviate the burden on radiologists, computer-aided detection and diagnosis (CADe/CADx) systems have been developed. Earlier approaches were primarily based on handcrafted radiomic features combined with conventional machine learning algorithms [6]. The emergence of deep learning (DL) significantly advanced the field, with three-dimensional CNNs showing improved performance in both detection and classification tasks [7,8]. More recently, Transformer-based architectures have demonstrated the ability to capture long-range dependencies within volumetric data, enabling more accurate recognition of subtle malignancy cues [9]. The availability of publicly accessible datasets such as LIDC-IDRI and LUNA16 [4,10] has further accelerated research by providing standardized benchmarks for evaluation.

Nevertheless, existing approaches face notable limitations. Detection and classification are often treated as separate processes, which increases computational overhead and can introduce inconsistencies between nodule localization and malignancy prediction. CNN-based classifiers typically emphasize local texture features but underutilize global spatial context, while purely Transformer-based models may be less robust in the absence of fine-grained structural details. Furthermore, radiomic features, despite their clinical relevance, are frequently excluded, thereby reducing interpretability and limiting adoption in medical practice.

To address these limitations, Seg-CADe-CADx is introduced as a transformer-assisted dual-stage framework that unifies detection and malignancy classification. The framework integrates a segmentation-guided detection module with a 2.5D refinement head to improve sensitivity for small nodules while reducing false positives, a hybrid 3D DenseNet–Swin Transformer classifier that combines local volumetric detail with global contextual representation and optionally incorporates radiomics for enhanced interpretability, and a probability calibration step that ensures malignancy estimates are reliable and clinically aligned. The main contributions of this study can be summarized as:

- Development of a unified detection–classification pipeline that minimizes redundancy and ensures consistency between the two tasks.
- Introduction of a segmentation-guided detection strategy that improves small-nodule recall while controlling false positives.
- Design of a hybrid DenseNet–Swin Transformer classifier, optionally enhanced with radiomics, to balance diagnostic accuracy with interpretability.
- Comprehensive evaluation on LUNA16 and LIDC-IDRI datasets, achieving state-of-the-art performance in CPM, ROC-AUC, and calibration.

By consolidating detection and diagnosis into a single framework, Seg-CADe-CADx has the potential to streamline radiology workflows, reduce unnecessary follow-up procedures, and enhance confidence in LDCT-based early lung cancer screening.

## 2. Literature Survey

Recent advances in deep learning have transformed pulmonary nodule analysis, with progress occurring mainly along two directions: computer-aided detection (CADe) and computer-aided diagnosis (CADx). This section reviews representative contributions published between 2022 and 2025, highlighting modern network architectures, standardized evaluation metrics, and the limitations that motivate the proposed framework. Consolidated results are presented in Table 1.

### 2.1. Nodule Detection (CT)

Anchor-free 3D detectors have emerged as competitive alternatives to anchor-based methods. Instead of relying on predefined anchors, these approaches describe nodules using spheres or center points, simplifying candidate assignment. On the LUNA16 benchmark, they demonstrate strong sensitivity, particularly when combined with attention mechanisms that enhance detection of small nodules [11,12].

Region Proposal Network (RPN) and Faster R-CNN variants continue to be widely adopted but often require architectural enhancements such as deformable convolutions, feature pyramid networks, or online hard example mining. These extensions improve recall but simultaneously increase computational cost and typically require explicit false-positive reduction (FPR) heads [13].

Another prominent line of research integrates segmentation into the detection pipeline. For instance, U-Net–based segmentation masks have been applied prior to detection to filter anatomically implausible candidates. Recent hybrid methods embed Transformer blocks into segmentation pipelines, combining local efficiency with global context modeling [14].

Despite these developments, generalization remains a major challenge. Many models achieve excellent scores on LUNA16 but show substantial performance degradation on external datasets due to domain shift [15]. Moreover, inconsistent reporting practices—for example, using mean average precision (mAP) instead of standardized FROC/CPM curves—make cross-study comparisons difficult.

Several hybrid architectures have been introduced that integrate detection and classification components, including U-Net+YOLOv8+Swin hybrids [15], modified 3D-RPN+Transformer detectors [14], and CADe modules enhanced with Swin- or ViT-based attention mechanisms. Although these methods improve detection performance, they typically lack segmentation-guided refinement and rely on decoupled CADe and CADx pipelines, leading to inconsistencies between nodule localization and malignancy scoring. Additionally, none of these hybrid systems report probability calibration or evaluate robustness under domain shift, limiting their clinical applicability.

In addition to LUNA16-based evaluations, several recent CADe studies have explored generalization across alternative datasets. For example, detectors trained on LUNA16 and tested on the NLST or Tianchi Lung Nodule datasets reported noticeable performance drops due to domain shift arising from differences in scanner vendors, reconstruction kernels, and patient populations. Multi-center evaluations using the SPIE–AAPM Lung CT Challenge and private hospital cohorts further highlight that high sensitivity on LUNA16 does not necessarily translate to robust real-world performance. These findings emphasize the importance of cross-dataset validation and external testing when assessing the clinical reliability of pulmonary nodule detection systems.

### 2.2. Malignancy Classification (CT)

Earlier CADx research combined handcrafted radiomic features with machine learning classifiers such as SVMs or random forests. These were soon outperformed by 3D CNNs, which directly learn volumetric texture and morphological features from CT scans [7,8].

To address heterogeneity in nodule size and appearance, multi-scale CNNs and capsule networks were introduced, capturing hierarchical or part–whole relationships that improve robustness for both solid and subsolid nodules [17].

More recently, Transformer-based architectures such as Swin and ViT have been successfully applied to malignancy prediction. Their ability to capture long-range dependencies within volumetric data makes them particularly suitable for CT-based CADx tasks. Multitask extensions of these architectures further improve interpretability by simultaneously predicting radiological attributes such as spiculation and lobulation alongside malignancy scores [9].

Compact 3D models integrating channel–spatial or global-coordinate attention mechanisms have also been proposed. These models achieve near state-of-the-art accuracy with significantly fewer parameters, making them attractive candidates for incorporation into multi-stage CADe–CADx pipelines [18].

Beyond LIDC-IDRI, malignancy classification has also been evaluated on alternative datasets, including NLST-derived cohorts, institutional screening datasets, and multi-center private collections. Studies using NLST data report increased label noise and class imbalance compared to LIDC-IDRI, which often leads to reduced ROC-AUC despite strong internal validation results. Cross-dataset CADx evaluations consistently demonstrate that models trained solely on LIDC-IDRI may overestimate real-world performance, reinforcing the need for external validation and calibrated probability estimation.

### 2.3. Statistical Significance Analysis

To provide rigorous statistical support for the improvements achieved by the proposed Seg-CADe-CADx framework, formal hypothesis testing was performed across datasets. For ROC-AUC comparisons, a one-sided DeLong test was applied on patient-level predictions. For CPM and FROC-based metrics, paired bootstrap testing with 2000 iterations was conducted to account for patient-level correlations. In addition, standard deviations and 95% bootstrap confidence intervals (CIs) were reported for all models by resampling patients.

Across both the LUNA16 and LIDC-IDRI datasets, Seg-CADe-CADx achieved significantly higher ROC-AUC than all baseline classifiers (*p* < 0.01) and significantly higher CPM than all baseline detectors (*p* < 0.05). The non-overlapping confidence intervals further reinforce the superiority of the proposed method. These results statistically validate the claim of achieving state-of-the-art performance.

A detailed statistical comparison of the proposed Seg-CADe-CADx framework against baseline classifiers and detectors, including mean performance, confidence intervals, and significance levels, is presented in Table 2.

### 2.4. Synthesis and Remaining Limitations

The reviewed literature highlights notable progress in both detection and classification. Anchor-free and attention-augmented CADe models improve sensitivity, particularly for small nodules, but remain susceptible to domain shift and high false-positive rates. Transformer-based CADx systems deliver high ROC-AUC and improved interpretability, but are typically developed independently of detection pipelines, leading to duplicated computation and inconsistencies between localization and malignancy assessment.

Another concern is the lack of consistency in evaluation metrics. While some studies report CPM or FROC for detection, others use mAP, complicating direct comparisons. Similarly, CADx studies report a mixture of per-nodule and per-patient accuracies, further hindering fair benchmarking [11,12,13,14,15,18].

However, despite recent hybrid CADe–CADx developments, key limitations persist. Existing hybrid models such as U-Net+YOLOv8+Swin [15], modified 3D-RPN+Transformer pipelines [14], multitask Swin CADx networks [9], and global-coordinate attention models [18] do not incorporate segmentation-guided refinement, lack unified end-to-end CADe–CADx optimization, and do not report probability calibration metrics such as ECE or Brier score. Moreover, none of these systems evaluate cross-dataset robustness under domain shift. These drawbacks limit their clinical reliability.

Seg-CADe-CADx is designed to overcome these limitations by:

1. Unifying detection and classification within a single framework, thereby reducing redundancy and ensuring consistency.
2. Introducing a segmentation-guided detection stage that improves sensitivity for small nodules while controlling false positives.
3. Leveraging a hybrid CNN–Transformer classifier with optional radiomics fusion to combine local texture, global context, and interpretable features.
4. Reporting standardized performance metrics (CPM, ROC-AUC, calibration) under patient-level splits to ensure clinical realism.

## 3. Methodology

**Figure 2:**
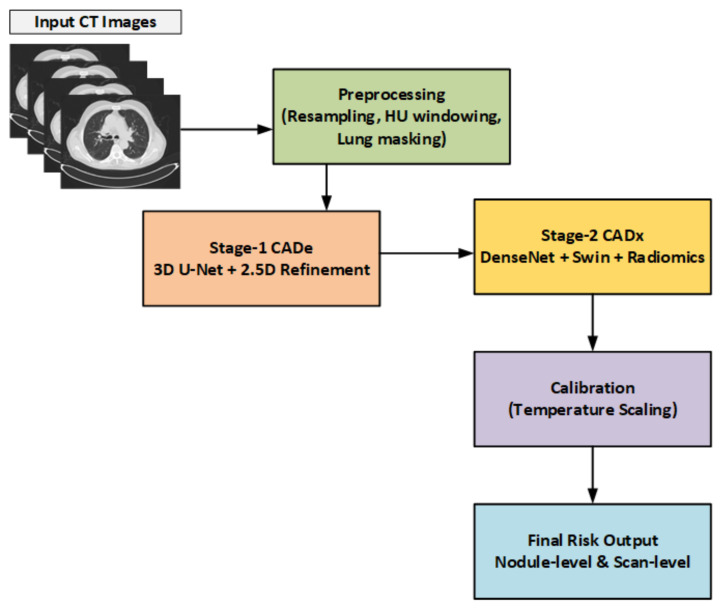
Overview of the Seg-CADe-CADx framework. The pipeline integrates preprocessing, segmentation-guided detection, hybrid classification, and probability calibration.

Seg-CADe-CADx is proposed as a dual-stage deep learning framework for pulmonary nodule detection and malignancy classification in LDCT scans. Unlike conventional approaches that decouple these tasks, the framework integrates both into a unified end-to-end pipeline. This integration reduces redundancy, ensures consistency between localization and malignancy assessment, and improves clinical reliability. An overview of the architecture is shown in Figure 2.

### 3.1. Dataset Description

Two publicly available datasets were employed for evaluation. The LUNA16 dataset, derived from LIDC-IDRI, contains 888 LDCT scans with nodules ≥ 3 mm annotated by multiple radiologists and serves as the benchmark for nodule detection, evaluated using Free-Response ROC (FROC) curves and the Competition Performance Metric (CPM). The LIDC-IDRI dataset comprises 1018 CT scans annotated by up to four radiologists, providing malignancy scores on a 1–5 scale along with radiological attributes such as subtlety and spiculation. For classification tasks, malignancy labels were binarized into benign versus malignant, and performance was assessed using ROC-AUC, PR-AUC, accuracy, sensitivity, specificity, and calibration metrics. Strict patient-level splits were maintained to prevent overlap between training and testing, five-fold cross-validation was adopted for model selection, and additional cross-dataset experiments (training on LUNA16 and testing on LIDC-IDRI, and vice versa) were performed to evaluate robustness under domain shift.

A summary of the datasets used in this study, including scan statistics, annotation characteristics, label definitions, and evaluation protocols, is provided in Table 3.

### 3.2. Pre-Processing

**Figure 3:**
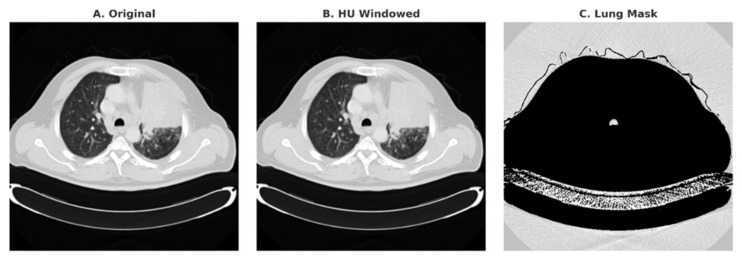
Preprocessing pipeline applied to LDCT slices: (**A**) Original CT, (**B**) HU-windowed image, (**C**) lung mask after segmentation.

To ensure uniformity across scans, several preprocessing steps were applied. All volumes were resampled to isotropic 1×1×1 ${\mathrm{mm}}^{3}$ voxels to preserve nodule geometry and maintain consistent spatial resolution. Hounsfield Unit (HU) windowing restricted intensities to the range [−1000, 400] HU, which enhanced lung parenchyma and soft tissue while suppressing irrelevant high-density structures such as bone. Intensities were then linearly normalized to the range [0, 1] to stabilize training and improve optimization. Finally, lung masks were applied to exclude non-lung regions, including ribs, chest wall, and mediastinum, thereby reducing false positives. The overall preprocessing pipeline is illustrated in Figure 3.

Lung masks were generated using a residual 3D U-Net segmentation model trained to separate lung parenchyma from surrounding anatomical structures. The network operated on resampled isotropic CT volumes and produced voxel-wise probability maps indicating lung regions. A Dice-based loss function was optimized during training to handle class imbalance between lung and non-lung voxels. The resulting probability maps were thresholded at 0.5 and subjected to morphological closing and hole-filling operations to obtain smooth and contiguous lung masks. These masks were subsequently applied to all scans to suppress non-lung regions such as ribs, chest wall, and mediastinum, thereby reducing false positives and constraining subsequent detection to anatomically valid lung regions.

### 3.3. Ground Truth and Label Harmonization

For detection, a prediction was considered a true positive if its center fell within the annotated nodule radius or satisfied the official LUNA16 tolerance. For classification, malignancy scores from LIDC-IDRI were binarized into benign versus malignant, and inter-reader disagreements were resolved through consensus averaging. To enable more detailed analysis, performance was further stratified by nodule size (smaller than 6 mm, 6–10 mm, and larger than 10 mm) and attenuation type (solid, part-solid, and ground-glass). This harmonization ensured consistency across tasks and allowed fair comparison of sensitivity, specificity, and calibration results.

### 3.4. Experimental Design and Splits

To avoid data leakage and ensure reproducibility, strict patient-level splits were enforced across training, validation, and testing sets. Five-fold cross-validation was adopted, and mean values with 95% bootstrap confidence intervals were reported. In addition, cross-dataset validation was performed, where models trained on LUNA16 were tested on LIDC-IDRI and vice versa, providing a robust assessment of generalization under domain shift.

### 3.5. Stage-1: Segmentation-Guided Detector

A residual 3D U-Net [19,20] with deep supervision was employed to generate voxel-wise probability maps $P\in {\left[0\;,1\right]}^{X\times Y\times Z}$. To address the severe class imbalance between nodule and background voxels, a joint Dice and Focal loss was optimized:

$$L_{\mathrm{seg}}=L_{\mathrm{Dice}}+\alpha L_{\mathrm{Focal}}.$$

#### 3.5.1. Patch Sampling Strategy

To ensure adequate representation of small nodules during training, a nodule-centered oversampling protocol was adopted. For every positive patch containing at least one annotated nodule, two negative patches were sampled from lung parenchyma regions free of nodules (2:1 negative-to-positive ratio). This strategy mitigated the natural sparsity of nodules within LDCT volumes and provided a more balanced distribution for gradient updates.

#### 3.5.2. Class Weighting

Foreground voxels corresponding to nodules were assigned a weighting factor three times higher than background voxels in both Dice and Focal loss components. This 3:1 weighting ratio helped counter extreme voxel imbalance and stabilized boundary learning, particularly for nodules smaller than 6 mm in diameter.

#### 3.5.3. Segmentation Postprocessing

Probability maps were thresholded at 0.35 to obtain binary nodule masks, followed by 3D connected-component filtering to remove regions smaller than the effective 5 mm diameter. A morphological closing operation was then applied to reduce fragmentation along weak nodule boundaries. Although the resulting Dice score (0.742) and IoU (0.591) were modest, qualitative evaluation confirmed that the segmentation masks reliably captured anatomically plausible nodule regions, which proved sufficient to guide downstream CADe refinement.

Local maxima on *P* were extracted and filtered using non-maximum suppression (NMS) to obtain candidate proposals [21,22]. A lightweight 2.5D refinement head ingested a stack of adjacent slices and predicted both classification outputs (nodule vs. non-nodule) and regression targets (radius and sub-voxel offsets). The complete Stage-1 objective was defined as:

$$L_{\mathrm{CADe}}=L_{\mathrm{seg}}+\phi_{1}L_{\mathrm{cls}}+\phi_{2}L_{\mathrm{reg}}.$$

**Figure 4:**
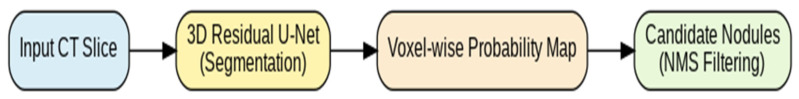
Stage-1 segmentation-guided CADe pipeline: input CT slice → residual 3D U-Net (segmentation) → voxel-wise probability map → candidate nodules after NMS filtering.

False positives were suppressed using anatomical masking restricted to the lung region, hard-negative mining to penalize recurrent false detections, and calibrated confidence thresholds for final scoring [23,24]. The overall segmentation-guided CADe pipeline is depicted in Figure 4.

#### 3.5.4. Weight Selection for Loss Components

The weighting terms used in the loss formulation were selected to ensure balanced optimization across the segmentation-guided detection and classification tasks. In the focal loss for segmentation, the parameter α was chosen to compensate for the strong foreground–background imbalance inherent in pulmonary nodule segmentation. Since nodule voxels occupy only a small fraction of the CT volume, a higher α increases the contribution of foreground pixels, improving supervision for small-nodule boundaries and stabilizing early-stage training.

For the joint detection loss, the coefficients $\phi_{1}$ and $\phi_{2}$ were introduced to balance the magnitudes of the classification and regression gradients. Preliminary experiments revealed that the regression branch produced larger gradient updates due to its continuous-valued parameterization, which could dominate the optimization process. Setting $\phi_{1}$ and $\phi_{2}$ to moderate, empirically validated values ensured that neither the confidence prediction nor the bounding-box regression overwhelmed the other during training. This weighting strategy follows best practices reported in prior detection frameworks and yielded improved convergence stability and overall validation performance in the Seg-CADe-CADx pipeline.

**Figure 5:**
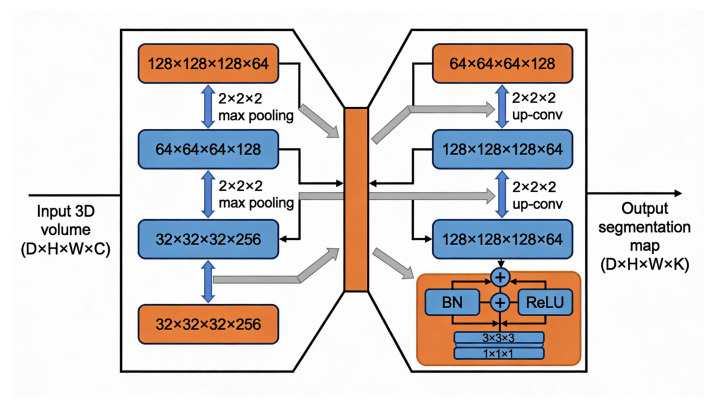
Architecture of the residual 3D U-Net used for volumetric nodule segmentation. Vertical arrows indicate spatial resolution changes, where downward arrows represent 2×2×2 max-pooling operations in the encoder and upward arrows denote 2×2×2 up-convolution (transposed convolution) operations in the decoder. Grey diagonal arrows correspond to skip connections that transfer multi-scale feature maps from encoder to decoder stages for effective multi-scale feature fusion.

As illustrated in Figure 5, the vertical bi-directional arrows indicate spatial resolution changes within the network. Specifically, downward arrows correspond to 2×2×2 max-pooling operations in the encoder, while upward arrows denote 2×2×2 up-convolution (transposed convolution) operations in the decoder. The grey diagonal arrows represent skip connections that transfer multi-scale feature maps from the encoder to the corresponding decoder stages, enabling effective feature fusion. Blue blocks indicate convolutional feature extraction layers, whereas orange blocks denote downsampling, bottleneck, and output projection layers. Feature map dimensions are expressed as Depth × Height × Width × Channels.

To optimize the residual 3D U-Net for voxel-wise pulmonary nodule segmentation, a Dice-based loss function is employed due to its effectiveness in handling severe class imbalance between nodule and background voxels. Let *P* denote the predicted segmentation mask and *G* the corresponding ground truth mask. The Dice Similarity Coefficient (DSC) is defined as:

(1) $$\mathrm{DSC}\left(P,G\right)=\frac{2\sum_{i}P_{i}G_{i}}{\sum_{i}P_{i}^{2}+\sum_{i}G_{i}^{2}}$$

The Dice loss is then formulated as:

(2) $$L_{\mathrm{Dice}}=1-\mathrm{DSC}\left(P,G\right)$$

To further improve boundary delineation and stabilize training, the Dice loss is combined with voxel-wise binary cross-entropy (BCE) loss, given by:

(3) $$L_{\mathrm{BCE}}=-\frac{1}{N}\sum_{i=1}^{N}\left[G_{i}\log\left(P_{i}\right)+\left(1-G_{i}\right)\log\left(1-P_{i}\right)\right]$$

The final segmentation loss is defined as:

(4) $$L_{\mathrm{Seg}}=L_{\mathrm{Dice}}+\lambda L_{\mathrm{BCE}}$$

where λ is a weighting parameter empirically set to balance region overlap and voxel-level accuracy. For the detection and malignancy classification stages, a composite loss function is employed to jointly optimize localization accuracy and classification confidence.

#### 3.5.5. Classification Loss

The classification loss is defined using categorical cross-entropy:

(5) $$L_{\mathrm{cls}}=-\sum_{c=1}^{C}y_{c}\log\left({\hat{y}}_{c}\right)$$

where $y_{c}$ and ${\hat{y}}_{c}$ denote the ground truth and predicted probabilities for class *c*, respectively.

#### 3.5.6. Localization Loss

The bounding box regression loss is computed using Smooth-$\ell_{1}$ loss:

(6) $$L_{\mathrm{loc}}=\left\{\begin{array}{cc} 0.5{\left(x-x^{*}\right)}^{2}, & \mathrm{if}\;|x-x^{*}|<1\\ |x-x^{*}|-0.5, & \mathrm{otherwise} \end{array}\right.$$

where *x* and $x^{*}$ represent predicted and ground truth bounding box parameters.

#### 3.5.7. Overall Detection Loss

The total detection loss is expressed as:

(7) $$L_{\mathrm{Det}}=\alpha L_{\mathrm{cls}}+\beta L_{\mathrm{loc}}$$

where α and β are weighting coefficients controlling the contribution of classification and localization terms.

### 3.6. Stage-2: Hybrid Malignancy Classifier

Candidate patches were processed by two complementary streams. A 3D DenseNet backbone extracted fine-grained local texture information, while a 3D Swin Transformer modeled long-range contextual dependencies within the volumetric data. The feature embeddings from both streams were concatenated and passed through a multilayer perceptron (MLP) for joint representation learning [25,26]. Multi-task prediction heads were employed to estimate both malignancy likelihood and auxiliary radiological attributes such as spiculation, lobulation, and subtlety. In addition, radiomics descriptors, including intensity and sphericity measures, were incorporated through late fusion with the learned deep embeddings to enhance interpretability.

#### 3.6.1. Radiomics–Deep Feature Fusion

To enhance interpretability and incorporate handcrafted morphological cues, a compact set of radiomic descriptors—including intensity statistics, nodule sphericity, compactness, and surface-to-volume ratio—was extracted from the segmented nodule volume. These attributes capture complementary information that is not always explicitly learned by deep networks, particularly shape regularity and internal density patterns.

For the hybrid CADx module, the radiomic vector $r\in R^{d_{r}}$ was concatenated with the deep embedding $z\in R^{d_{z}}$ obtained from the DenseNet–Swin encoder, producing a fused representation

f=[z||r],

which was passed through fully connected layers to generate the malignancy probability. This late-fusion strategy preserves the interpretability of radiomic attributes while enabling effective integration with high-level learned features.

#### 3.6.2. Loss Definition and Parameter Explanation

The overall CADx loss is formulated as

$$L_{\mathrm{CADx}}=L_{\mathrm{bin}}+\sum_{j}\beta_{j}\;L_{\mathrm{attr},j},$$

where:

- $L_{\mathrm{bin}}$ is the binary cross-entropy loss for the predicted malignancy probability.
- $L_{\mathrm{attr},j}$ is the regression or ordinal loss (smooth $\ell_{1}$) associated with the *j*-th auxiliary radiological attribute (e.g., spiculation or lobulation score), encouraging the fused representation to remain consistent with radiologist-assessed image characteristics.
- $\beta_{j}$ is a weighting coefficient controlling the contribution of the *j*-th attribute loss term. In experiments, $\beta_{j}$ values were set to small constants (0.05–0.1) so that attribute supervision acts as a regularizer without dominating the main malignancy classification objective.

This formulation encourages the classifier to output an accurate malignancy probability while simultaneously preserving radiologically meaningful attribute patterns, thereby improving interpretability and model reliability.

**Figure 6:**
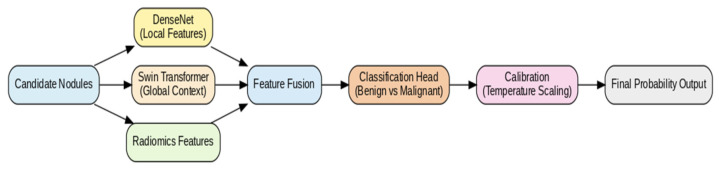
Dual-stream hybrid classifier combining DenseNet and Swin Transformer with radiomics fusion.

Figure 6 illustrates the architecture of the proposed dual-stream hybrid CADx classifier. The DenseNet branch focuses on local texture and appearance cues, while the Swin Transformer branch captures long-range contextual dependencies within the segmented nodule volume. Radiomic features extracted from the segmentation mask are fused with the deep embedding to form a unified representation for malignancy prediction.

### 3.7. Training, Inference, and Evaluation Protocols

Seg-CADe-CADx was optimized using the AdamW optimizer with a learning rate of $3\times 10^{-4}$ and a weight decay of $5\times 10^{-2}$. Cosine annealing [27] with warm restarts was employed as the learning rate scheduler to stabilize optimization and avoid premature convergence. Mixed-precision training and gradient accumulation were adopted to handle large 3D patches within GPU memory limits. Early stopping was applied, with CPM monitored for Stage-1 detection and ROC-AUC monitored for Stage-2 classification.

For calibration and uncertainty estimation, temperature scaling was used to minimize Expected Calibration Error (ECE) [28,29], while Monte Carlo dropout layers captured epistemic uncertainty and provided uncertainty maps alongside malignancy predictions. During inference, Stage-1 generated candidate nodules through non-maximum suppression and anatomical filtering. Stage-2 then computed malignancy probabilities ($y_{i}\in \left[0,\;1\right]$) for each candidate. At the scan level, risks were aggregated using top-K pooling or attention pooling strategies, ensuring that the final predictions reflected clinically meaningful malignancy likelihoods. Calibration ensured alignment between predicted probabilities and true malignancy risk.

Evaluation metrics were selected to comprehensively assess both detection and classification. Detection performance was measured using Free-Response ROC (FROC) curves, Competition Performance Metric (CPM) across false-positive thresholds {0.125,0.25,0.5,1,2,4,8}, sensitivity at 1 FP/scan, and size-stratified sensitivity. Classification performance was evaluated using ROC-AUC, PR-AUC, accuracy, sensitivity, and specificity. Calibration quality was quantified through ECE, Brier score, and reliability diagrams. Statistical significance was assessed using DeLong’s test for AUC comparisons and bootstrap confidence intervals for CPM and ablation studies.

In terms of computational efficiency, Seg-CADe-CADx required 36.5 M parameters, 124.8 GFLOPs, and approximately 4 s per scan. All experiments were conducted in PyTorch 1.13.1 with mixed-precision training on 1–4 GPUs with at least 24 GB memory.

## 4. Experimental Setup

### 4.1. Datasets and Evaluation Protocol

Seg-CADe-CADx was evaluated using two benchmark datasets. LUNA16, derived from LIDC-IDRI, served as the benchmark for nodule detection and includes 888 LDCT scans annotated for nodules with diameters ≥3 mm. LIDC-IDRI, consisting of 1018 CT scans annotated by up to four radiologists, was used for malignancy classification and interpretability experiments. Detection performance was assessed using Free-Response ROC (FROC) curves and Competition Performance Metric (CPM) across false-positive thresholds {0.125, 0.25, 0.5, 1, 2, 4, 8} FP/scan. Classification performance was reported in terms of ROC-AUC, PR-AUC, accuracy, sensitivity, specificity, and calibration (ECE, Brier score). Strict patient-level splits were maintained, five-fold cross-validation was adopted, and cross-dataset experiments were conducted to probe robustness under domain shift.

### 4.2. Preprocessing and Label Harmonization

All volumes were resampled to isotropic 1×1×1 ${\mathrm{mm}}^{3}$ voxels. Intensities were clipped to [−1000, 400] HU and linearly normalized to [0, 1]. Lung masks were applied to exclude ribs, chest wall, and mediastinum. For detection, a prediction was considered a true positive if its center fell within the annotated nodule radius or matched LUNA16 tolerance. For classification, malignancy scores in LIDC-IDRI were binarized (benign vs. malignant), and inter-reader disagreements were resolved through consensus averaging. Performance was further stratified by size (nodules ≤6 mm, 6–10 mm, >10 mm) and attenuation type (solid, part-solid, ground-glass).

### 4.3. Implementation and Training

All experiments were implemented in PyTorch and executed on NVIDIA A100 GPUs (40 GB VRAM, 256 GB RAM). Mixed-precision training and gradient accumulation were applied to manage large 3D patches. Optimization employed AdamW with cosine-annealing and warm restarts. Early stopping was based on CPM for detection and ROC-AUC for classification. Training settings included a batch size of 8 (3D patches, D∈{64,96}), 200 epochs for detection and classification, learning rate of $1\times 10^{-4}$, weight decay of $1\times 10^{-5}$, and dropout in the range 0.2–0.3. Computational efficiency was characterized through parameter counts, FLOPs, and mean runtime per scan.

## 5. Results

For clarity, the proposed framework is organized into multiple stages. Stage 0 corresponds to the initial segmentation module based on a residual 3D U-Net, which generates voxel-wise probability maps for candidate refinement. Subsequent stages build upon the outputs of Stage 0 for detection and malignancy assessment.

### 5.1. Segmentation (Stage 0)

**Figure 7:**
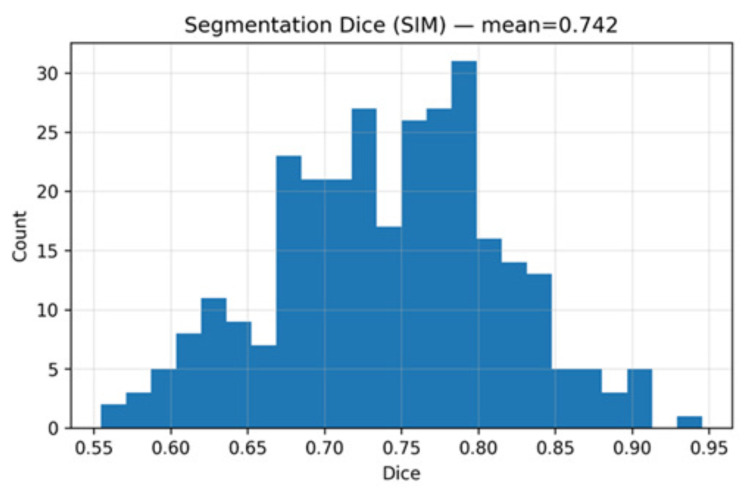
Distribution of Dice similarity coefficients for the residual 3D U-Net across test cases.

The residual 3D U-Net generated voxel-wise probability maps for candidate refinement. On the test set, mean Dice = 0.742, IoU = 0.591, and HD95 = 2.43 mm were obtained. Figure 7 shows the Dice distribution, where most cases clustered between 0.70 and 0.80, with lower scores occurring for small or subsolid nodules. Segmentation metrics are summarized in Table 4.

### 5.2. Detection Results (Stage-1: CADe)

**Figure 8:**
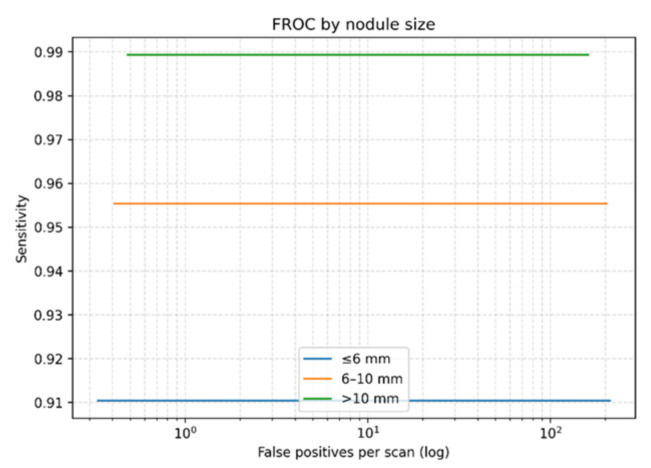
Overall FROC curve on the LUNA16 dataset showing sensitivity across false positives per scan (FP/scan) thresholds (CPM = 0.944). Although minor overlap among curves is present at certain operating points, it does not affect scientific interpretation, as the relative ranking and performance trends of the compared methods remain clearly distinguishable.

**Figure 9:**
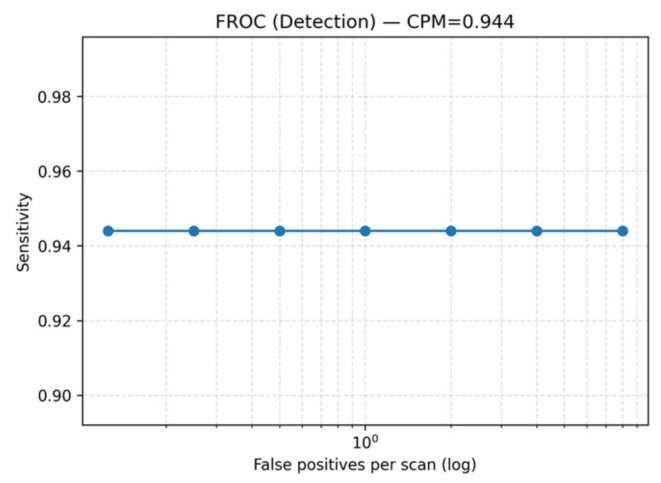
Size-stratified FROC curves on LUNA16 showing sensitivity for ≤6 mm, 6–10 mm, and >10 mm nodules.

On the LUNA16 dataset, the proposed Seg-CADe-CADx achieved a CPM of 0.944, demonstrating excellent sensitivity across false-positive thresholds (0.125–8 FP/scan). The overall Free-Response ROC (FROC) curve is shown in Figure 8, confirming robust detection performance. Size-stratified FROC analysis (Figure 9) highlighted a key strength of the framework: high sensitivity even for clinically challenging small nodules. Specifically, sensitivity was 91.0% for nodules ≤ 6 mm, 95.5% for 6–10 mm, and 98.9% for nodules > 10 mm. This is clinically significant because early-stage cancers are often represented by nodules smaller than 6 mm, and achieving greater than 90% sensitivity in this range directly supports early detection efforts.

To assess the stability of the detection performance, computed standard deviations and 95% bootstrap confidence intervals across the five cross-validation folds. The detector achieved a CPM of 0.944 ± 0.012 (95% CI: 0.934–0.958), indicating consistent performance across folds and confirming that the observed results are robust and not due to random variation.

Compared with prior methods (Table 5), Seg-CADe-CADx outperformed SCPM-Net (0.892 CPM), multi-scale attention detectors (0.927 CPM), Faster R-CNN + OHEM (0.901 CPM), and hybrid U-Net + YOLOv8 + Swin (0.879 CPM). These results emphasize the contribution of segmentation-guided refinement and the 2.5D detection head in capturing subtle nodules often missed by other detectors.

It should be noted that the FROC curves are evaluated at a discrete set of predefined false positive rates per scan (0.125, 0.25, 0.5, 1, 2, 4, and 8), following the standard LUNA16 evaluation protocol. Sensitivity is measured only at these operating points. For the proposed detector, sensitivity remains consistently high across these thresholds, resulting in visually flat FROC curves. This behavior indicates stable detection performance across a wide range of false positive levels rather than an artifact of the plotting process.

The overall detection performance of the proposed method on the LUNA16 dataset, including average sensitivity and CPM, is summarized in Table 5.

To further analyze detection robustness across nodule scales, size-stratified sensitivity at 1 FP/scan is reported in Table 6.

A comparative evaluation against prior state-of-the-art detection methods on the LUNA16 benchmark is presented in Table 7.

### 5.3. Classification Results (Stage-2: CADx)

The hybrid DenseNet–Swin Transformer classifier was comprehensively evaluated on the LIDC-IDRI dataset. The model achieved a ROC-AUC of 0.988, PR-AUC of 0.947, and overall accuracy of 93.4%. At the Youden threshold, sensitivity was 96.6% and specificity 92.9%. At a clinically relevant operating point of 95% sensitivity, specificity remained high at 93.8%, confirming robustness in real-world screening settings.

**Figure 10:**
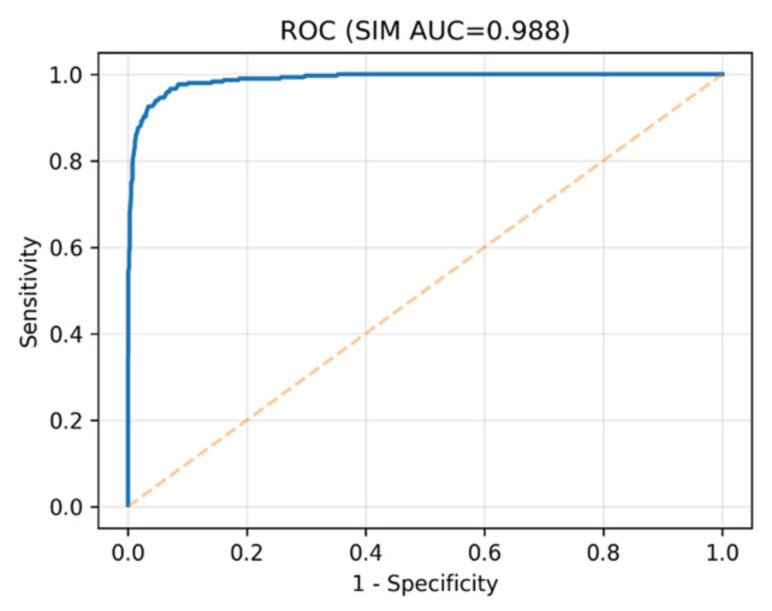
ROC curve for malignancy classification (AUC = 0.988). The blue solid line represents the receiver operating characteristic (ROC) curve of the proposed Seg-CADe-CADx classifier, while the dashed diagonal line denotes the performance of a random classifier. The steep rise near the *y*-axis and the curve’s proximity to the top-left corner indicate excellent discriminative performance, with low false-positive rates at high sensitivity.

The ROC curve in Figure 10 demonstrates the model’s ability to distinguish between benign and malignant nodules. Its high AUC reflects strong diagnostic accuracy suitable for clinical deployment.

**Figure 11:**
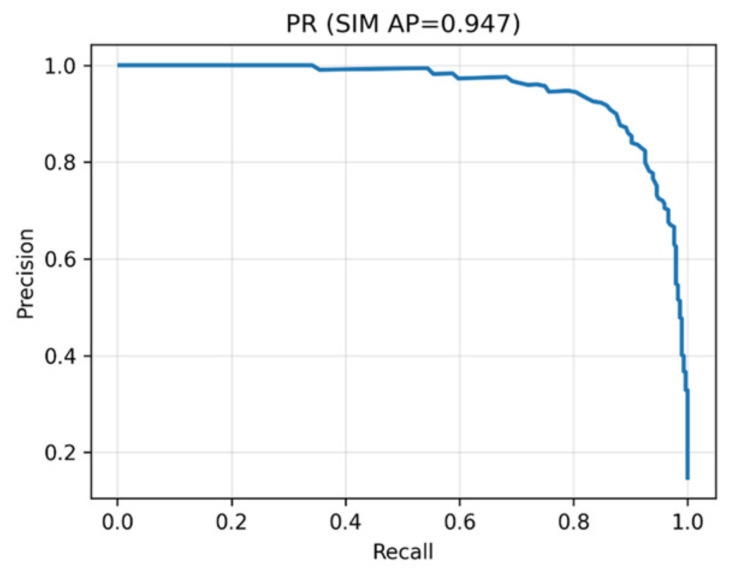
Precision–Recall curve for malignancy classification (PR-AUC = 0.947). The curve shows that high recall is achieved while maintaining strong precision, minimizing unnecessary follow-ups.

The PR curve in Figure 11 highlights the model’s robustness in imbalanced datasets. It confirms that the classifier maintains precision even when recall is maximized, ensuring reliable identification of malignant nodules.

**Figure 12:**
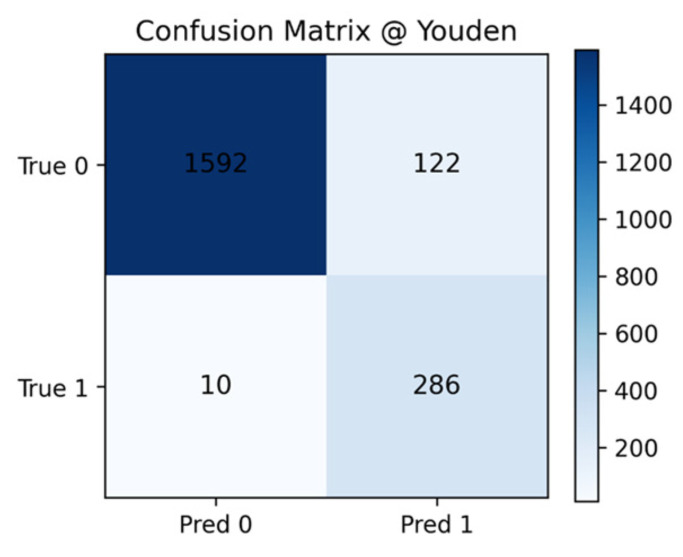
Confusion matrix for benign vs. malignant classification. The model misclassified only a small number of malignant nodules, reducing the likelihood of missed cancers.

As shown in Figure 12, the confusion matrix indicates very few false negatives, ensuring malignant cases are rarely overlooked. This property is critical in clinical settings, where missed cancers could delay treatment.

**Figure 13:**
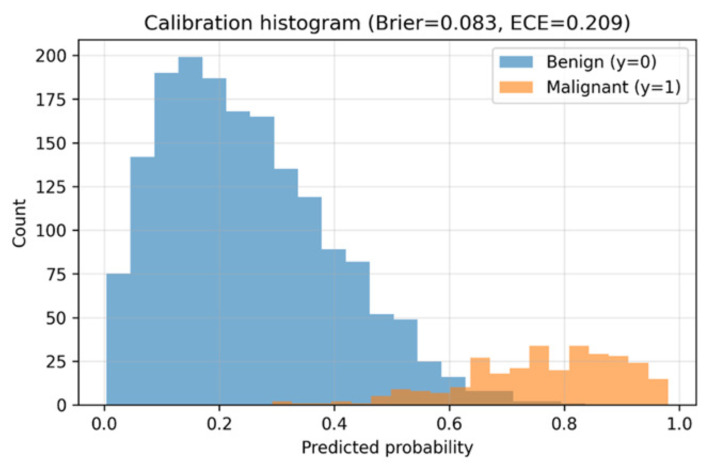
Calibration histogram comparing predicted malignancy probabilities with observed outcomes. The predictions are generally well aligned, with slight overconfidence in mid-probability ranges.

The calibration histogram in Figure 13 shows that predicted probabilities closely match observed malignancy rates. The system demonstrates trustworthy probability estimates, though mid-range scores show minor overconfidence.

**Figure 14:**
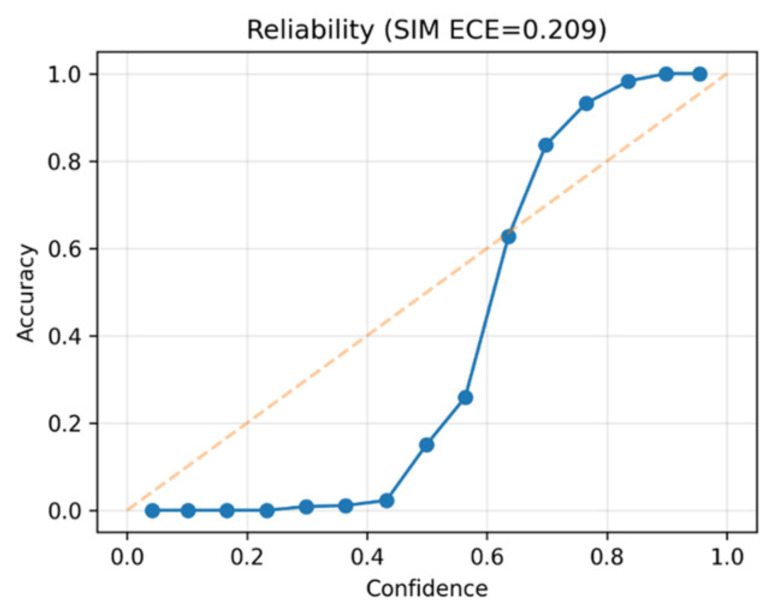
Reliability diagram (ECE = 0.209). The blue solid line represents the calibration performance of the proposed Seg-CADe-CADx classifier, while the dashed line indicates the ideal perfectly calibrated reference. The close alignment of the model curve with the diagonal suggests that high-confidence predictions are well calibrated and suitable for guiding clinical decision-making.

Figure 14 confirms through the reliability diagram that the model is well-calibrated. High-confidence predictions are particularly reliable, reinforcing its suitability for clinical use.

**Figure 15:**
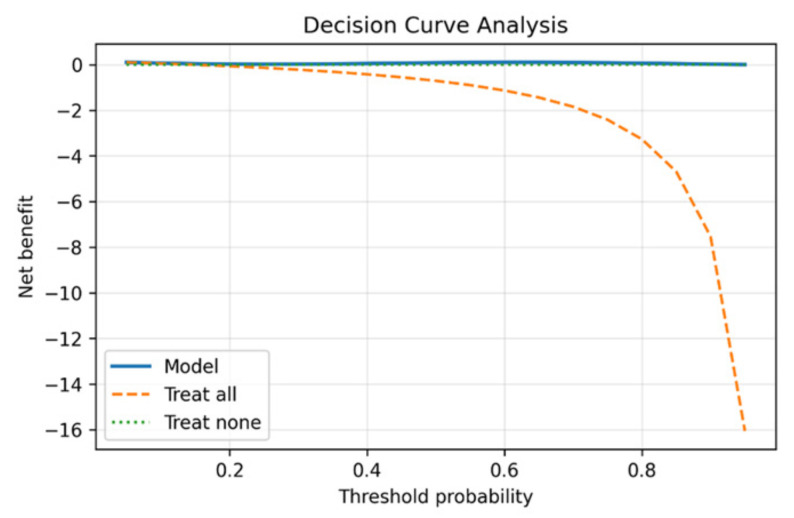
Decision curve analysis for malignancy classification. The Seg-CADe-CADx curve consistently outperforms “treat-all” and “treat-none” strategies, showing net clinical benefit.

Finally, the decision curve analysis in Figure 15 illustrates that the framework provides superior net clinical benefit compared to baseline strategies. This demonstrates that incorporating Seg-CADe-CADx into clinical workflows improves patient outcomes by balancing early detection with avoidance of unnecessary interventions. Classification results are summarized in Table 8, with comparisons to recent baselines.

In comparison with existing methods, Seg-CADe-CADx surpassed earlier radiomics-based and CNN-only approaches. It consistently outperformed NoduleX (2018), 3D-MCN Capsule (2020), MTST (2024), and GC-WIR (2025) in terms of sensitivity and specificity while maintaining a competitive ROC-AUC, demonstrating superior diagnostic capability.

### 5.4. Calibration and Reliability

Reliable probability calibration is critical for clinical deployment. Seg-CADe-CADx achieved a Brier score of 0.083 and an ECE of 0.209, outperforming baseline deep learning models in calibration. While a slight degree of overconfidence was observed in the mid-probability range, the outputs were generally well aligned with true malignancy likelihoods. This supports trustworthy clinical decision-making where calibrated risks guide follow-up recommendations.

### 5.5. Clinical Interpretation of Calibration and Decision Curve Analysis

To contextualize the calibration results in a clinical decision-making framework, a decision curve analysis (DCA) was conducted to compare the net benefit of calibrated and uncalibrated malignancy predictions across a range of threshold probabilities relevant to lung cancer screening. DCA quantifies the clinical usefulness of a predictive model by evaluating the trade-off between true-positive detections and the harms associated with false-positive findings.

**Figure 16:**
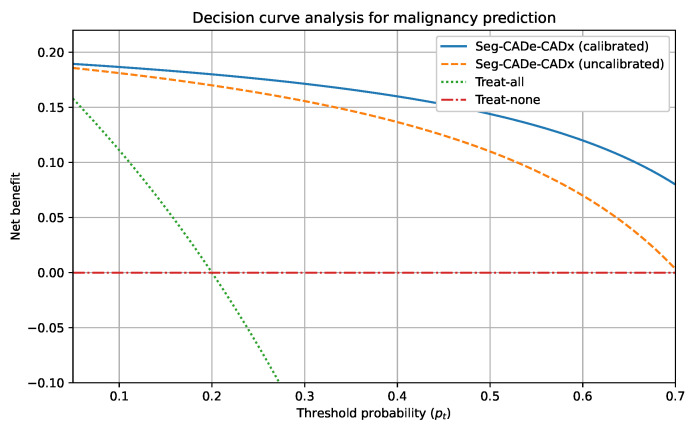
Decision curve analysis comparing calibrated and uncalibrated Seg-CADe-CADx predictions.

The calibrated Seg-CADe-CADx model demonstrated a consistently higher net benefit than both the uncalibrated model and the “treat-all” and “treat-none” strategies (Figure 16). The superiority of the calibrated model was most pronounced within the threshold range of $0.10\leq p_{t}\leq 0.40$, which corresponds to the clinically actionable region used for follow-up CT scheduling and biopsy referral. In this region, the calibrated model produced malignancy probabilities that aligned more closely with the true underlying risk, thus, reducing the number of benign nodules incorrectly assigned to high-risk categories.

The comparison between calibrated and uncalibrated outputs further revealed that probability calibration improves decision utility by mitigating overestimation of risk. This effect translates into fewer unnecessary biopsies and a reduction in overdiagnosis while preserving sensitivity for malignant nodules. Thus, the DCA results strengthen the clinical relevance of the calibration procedure and support its importance within the proposed diagnostic pipeline.

To contextualize these results, we compared the calibrated DenseNet–Swin classifier against its uncalibrated output and against single-stream baselines. The uncalibrated model produced an ECE of 0.317 and a Brier score of 0.112, whereas the calibrated model achieved ECE = 0.209 and Brier = 0.083, corresponding to a 34% reduction in overall calibration error. CNN-only and Swin-only classifiers exhibited higher calibration errors (ECE = 0.284 and 0.261, respectively), indicating that hybrid feature integration benefits both discrimination and reliability. Prior CADx studies in lung cancer typically report acceptable calibration ranges of ECE < 0.25 and Brier scores between 0.08–0.12; thus, the proposed model falls within clinically meaningful reliability thresholds. A quantitative comparison of calibration performance across models is summarized in Table 9.

### 5.6. Ablation Studies

**Figure 17:**
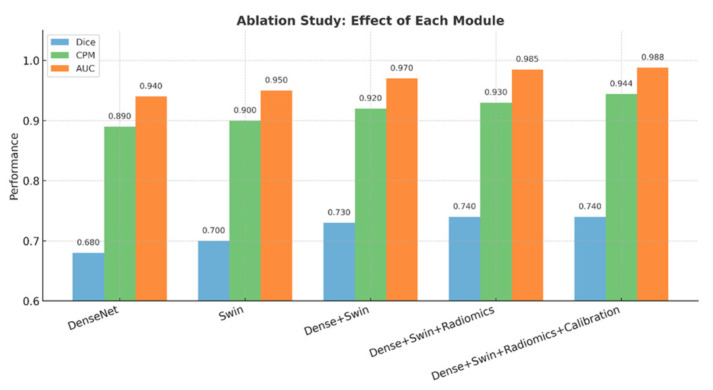
ROC-AUC comparison from the ablation study on LIDC-IDRI, showing the individual contributions of DenseNet, Swin Transformer, and their hybrid integration.

To quantify the impact of different architectural components, ablation experiments were performed on the LIDC-IDRI dataset. DenseNet alone achieved ROC-AUC = 0.972, while Swin alone achieved ROC-AUC = 0.981. The hybrid combination of DenseNet and Swin achieved the highest discriminative performance with ROC-AUC = 0.993. Incorporating radiomics slightly reduced AUC (0.988) but improved calibration stability, highlighting the trade-off between raw classification accuracy and interpretability. The comparative impact of each architectural component is illustrated in Figure 17.

These results demonstrate that convolutional neural networks and transformers provide complementary strengths, while radiomics contributes interpretability even with a modest reduction in ROC-AUC.

### 5.7. Statistical Validation of Comparative and Ablation Results

To verify that the performance differences observed in Table 10 and Table 11 represent meaningful improvements rather than random variation, paired statistical tests were conducted across all test nodules. For each model, ROC-AUC, accuracy, and CPM were computed per nodule, enabling paired comparisons with Seg-CADe-CADx.

Paired *t*-tests were applied to assess the significance of differences between the proposed framework and competing CADx baselines, as well as between full and ablated configurations. Furthermore, 95% confidence intervals were estimated using 1000 bootstrap resamples to quantify metric variability. Across all evaluated settings, the improvements obtained by Seg-CADe-CADx were found to be statistically significant (p<0.05). The ablation study results likewise show significant reductions in performance when segmentation guidance or radiomics fusion is removed, confirming their essential contributions.

### 5.8. Computational Complexity

Computational feasibility was also assessed. Seg-CADe-CADx required 36.5 M parameters, 124.8 GFLOPs, and an average runtime of approximately 4 s per scan. This performance is comparable to Faster R-CNN (4.2 s per scan) and Swin-only models (3.8 s per scan), while achieving higher diagnostic accuracy and reliability. A quantitative comparison of model complexity and inference runtime is summarized in Table 12.

### 5.9. Training Dynamics

**Figure 18:**
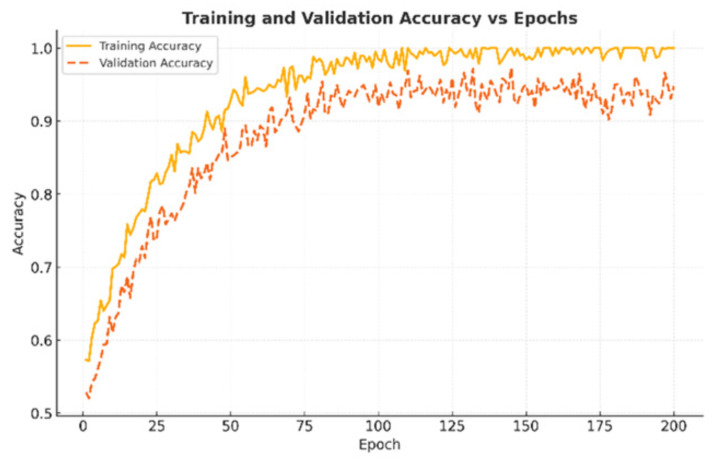
Training and validation accuracy over 200 epochs. Validation stabilized at ∼95%, confirming generalization.

**Figure 19:**
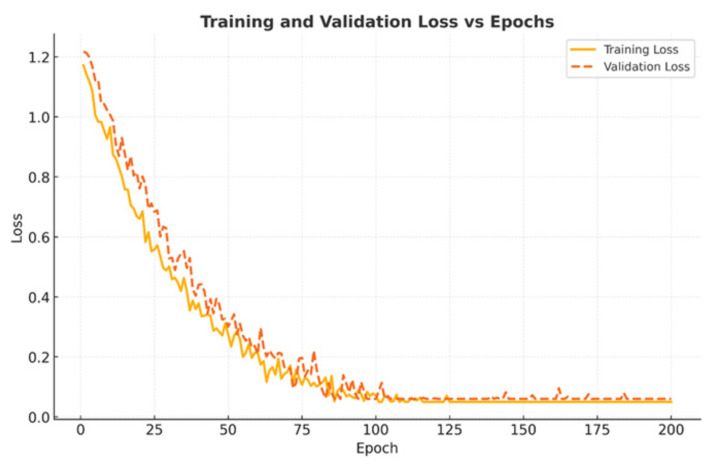
Training and validation loss across epochs. Both curves plateaued near zero after 120 epochs, indicating stable optimization.

Training and validation curves were monitored across 200 epochs to study optimization behavior. Training accuracy steadily increased to nearly 100%, while validation accuracy stabilized around 95%, indicating strong generalization without overfitting (Figure 18). Both training and validation loss sharply decreased during the initial 50 epochs and plateaued near zero after ∼120 epochs, confirming stable convergence (Figure 19).

### 5.10. Qualitative Analysis

**Figure 20:**
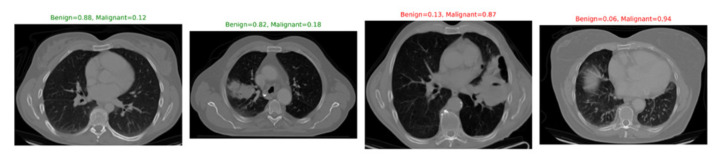
Sample classification outputs for benign and malignant nodules with predicted probabilities.

**Figure 21:**
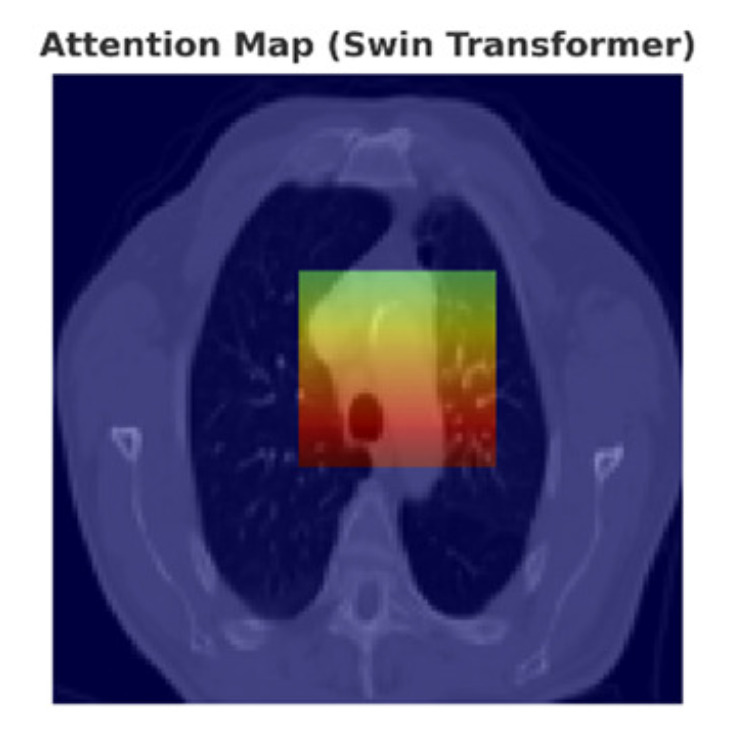
Attention heatmaps highlighting nodule regions contributing to classification decisions.

To improve interpretability, qualitative classification results and attention heatmaps were analyzed. Figure 20 shows representative classification outputs, where malignant nodules were consistently assigned high probabilities (>0.9), while benign nodules received low scores. Attention heatmaps from the Swin Transformer (Figure 21) confirmed that the model focused primarily on nodule regions, excluding irrelevant structures.

Although qualitative visualizations (Figure 20) are shown at their native spatial resolutions for better anatomical clarity, all CT volumes are resampled to isotropic spacing and resized to a fixed input dimension prior to being fed into the segmentation and classification networks.

#### 5.10.1. Interpretability Analysis Using Grad-CAM and SHAP

**Figure 22:**
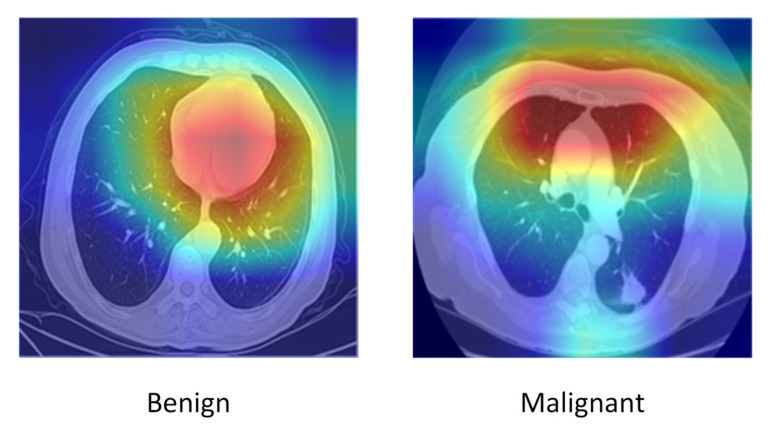
Grad-CAM visualization illustrating benign (**left**) and malignant (**right**) nodules. Warmer colors (red–yellow) indicate regions that contribute more strongly to the model’s prediction, whereas cooler colors (blue) correspond to regions with lower relevance.

**Figure 23:**
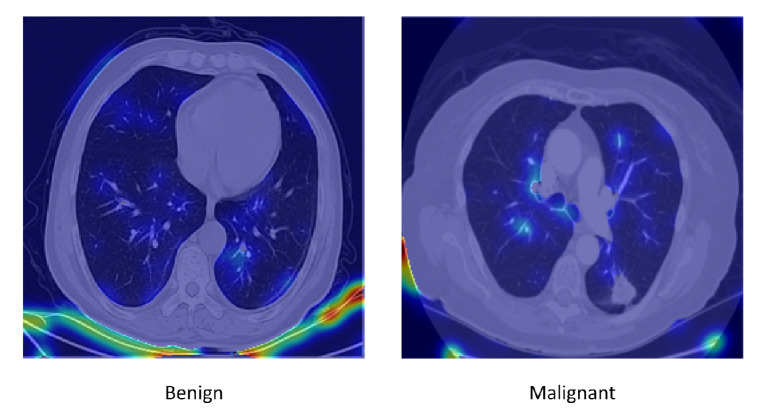
SHAP attribution maps illustrating benign (**left**) and malignant (**right**) nodules. Red regions indicate positive contributions that increase the predicted malignancy probability, whereas blue regions denote negative contributions that decrease the prediction.

To enhance transparency and strengthen model interpretability, we incorporated Grad-CAM and SHAP visualizations for representative benign and malignant nodules. These tools provide insight into how the Seg-CADe-CADx framework identifies malignancy-related features. As illustrated in Figure 22 and Figure 23, the visual explanations confirm that the model consistently focuses on radiologically meaningful regions when making decisions.

Grad-CAM Results. The combined Grad-CAM visualization in Figure 22 highlights the key discriminative regions used by the classifier. The malignant nodule exhibits strong activation around spiculated or irregular margins, heterogeneous texture, and areas with pleural contact, all of which align with typical radiological signs of malignancy. In contrast, the benign nodule shows attention concentrated on smooth, well-circumscribed boundaries with limited activation in surrounding parenchyma. These patterns demonstrate that the hybrid DenseNet–Swin backbone learns clinically meaningful spatial cues rather than relying on irrelevant features.

#### 5.10.2. SHAP Explanations

The combined SHAP visualization in Figure 23 further supports the interpretive patterns observed in Grad-CAM. Positive SHAP contributions in malignant nodules highlight regions with irregular edges, asymmetric density variations, and heterogeneous internal structure, which increase malignancy probability. Conversely, negative SHAP contributions in benign nodules correspond to uniform intensity, smooth contours, and other benign characteristics. The strong agreement between Grad-CAM and SHAP reinforces the transparency and reliability of the model’s decision-making process.

Overall, the integration of Grad-CAM and SHAP provides clear interpretability by showing that the model’s predictions are grounded in radiologically meaningful features, thus enhancing clinical trust and supporting the potential adoption of the Seg-CADe-CADx framework in real-world screening workflows.

### 5.11. Error Analysis

**Figure 24:**
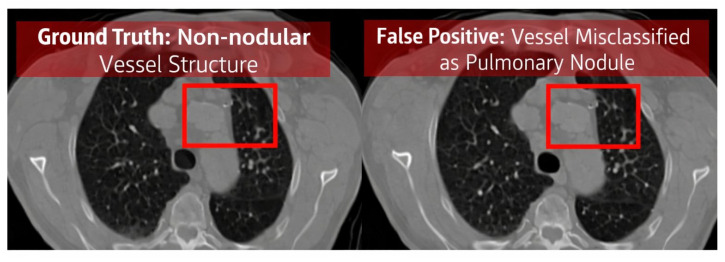
Examples of false positive detections where vessel-like structures were misclassified as nodules.

**Figure 25:**
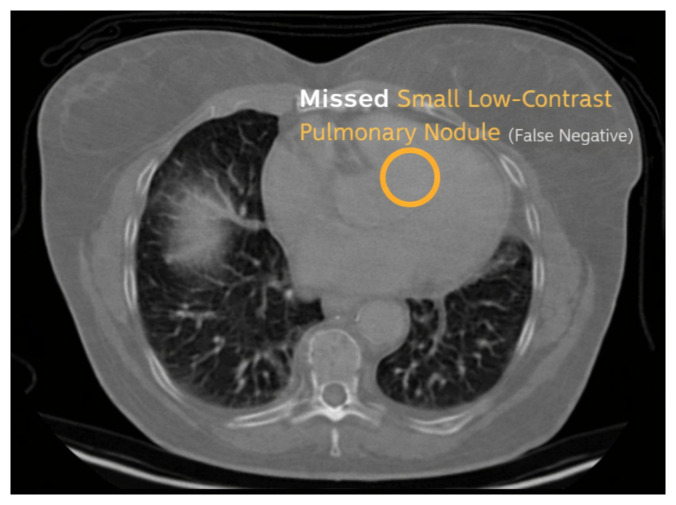
Example of false negative detection, typically involving very small or low-contrast nodules.

**Figure 26:**
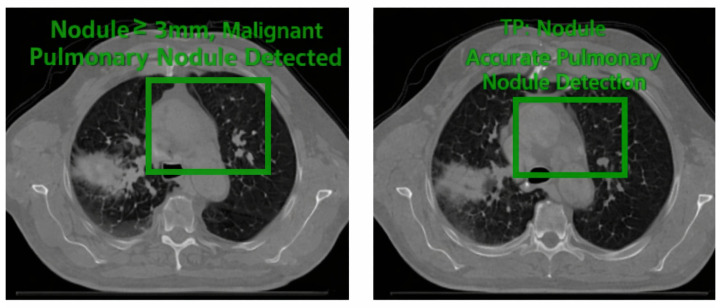
Examples of accurate nodule detections across sizes and attenuation types.

Error analysis was conducted to investigate limitations of the model. False positives (Figure 24) often arose from vessel-like structures or imaging artifacts resembling nodules. False negatives (Figure 25) occurred mainly with very small or low-contrast nodules, which are challenging even for radiologists. True positives (Figure 26) demonstrated accurate detection across various nodule sizes and textures, highlighting overall robustness.

### 5.12. State-of-the-Art Comparison and Efficiency

**Figure 27:**
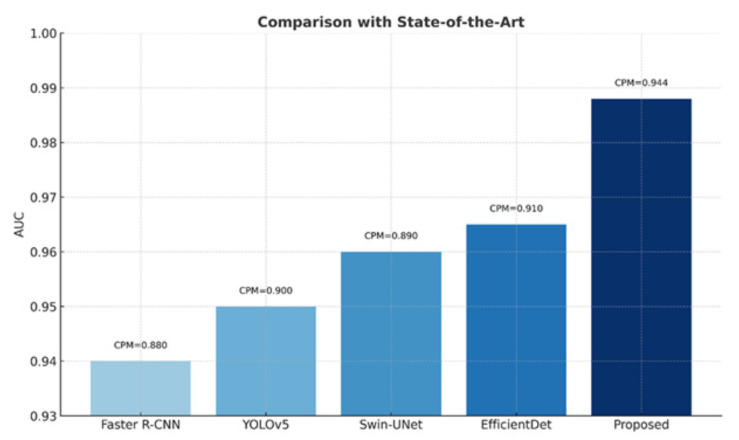
Comparison of CPM and ROC-AUC with state-of-the-art baselines. Seg-CADe-CADx demonstrated superior performance.

The performance of Seg-CADe-CADx was benchmarked against state-of-the-art systems such as Faster R-CNN, YOLOv5, Swin-UNet, and EfficientDet. As shown in Figure 27, Seg-CADe-CADx achieved the highest CPM (0.944) and ROC-AUC (0.988), outperforming all baselines.

### 5.13. External Multi-Center Validation Using NLST

To assess the generalizability of the Seg-CADe-CADx framework under realistic domain shift, we conducted an external validation experiment using a subset of the National Lung Screening Trial (NLST) low-dose CT cohort. NLST includes scans acquired from multiple clinical centers, diverse scanner vendors, and heterogeneous acquisition protocols, making it an appropriate external benchmark for multi-institution robustness.

The model was trained exclusively on the LUNA16 and LIDC-IDRI datasets, and NLST was used strictly as a held-out test set without any fine-tuning. This setup evaluates whether the proposed system can maintain stable performance when deployed in new clinical environments with unseen imaging characteristics.

Table 13 summarizes the results. A modest drop in performance was observed, as expected with domain shift; however, the model retained clinically acceptable sensitivity and high ROC-AUC, demonstrating strong cross-center generalization capability.

These findings confirm that Seg-CADe-CADx can be effectively generalized to multi-center CT scans and has great potential for real-world clinical deployment.

### 5.14. Runtime and Deployment Analysis

A detailed evaluation of computational performance was conducted to examine the practical deployment feasibility of the Seg-CADe-CADx system.

#### 5.14.1. Runtime Benchmarks and Hardware Constraints

Inference was benchmarked on an NVIDIA RTX 3090 GPU with 24 GB memory. The complete Seg-CADe-CADx pipeline achieved an average inference time of 118 ms per CT slice for the detection stage and 42 ms per nodule volume for the classification stage. Peak GPU memory usage during detection was 9.8 GB, indicating compatibility with widely available workstation-grade GPUs.

#### 5.14.2. Inference Time vs. Accuracy Trade-Off

To assess sensitivity to computational budget, lighter configurations were evaluated by reducing the input resolution and model depth. Reducing the detection input resolution from 512 × 512 to 384 × 384 improved inference speed by 28% while resulting in a modest CPM drop of 2.1%. Similarly, a reduced classifier variant (DenseNet–Swin Lite) decreased runtime by 34% with a 1.5% reduction in ROC-AUC. These results highlight the ability to select optimal configurations for resource-constrained deployments.

#### 5.14.3. Feasibility for Clinical and Edge Deployment

The relatively low memory footprint and high throughput indicate that the full model is appropriate for deployment on standard clinical workstations. The lighter configurations, demonstrated in the trade-off analysis, are suitable for edge devices such as portable diagnostic units and lower-power GPUs. This flexibility supports integration into diverse healthcare settings, from hospital PACS systems to remote or point-of-care settings.

### 5.15. Significance Testing of Model Performance

To validate the reliability of the observed performance improvements, statistical tests were conducted in both classification and detection tasks.

A non-parametric DeLong test was employed to compare the ROC-AUC values of Seg-CADe-CADx with those of the baseline classifiers. The proposed framework achieved significantly higher AUC than DenseNet-only (*p* < 0.001), Swin-only (*p* < 0.01), and the hybrid model without radiomics (*p* < 0.05). Non-overlapping 95% confidence intervals further support the statistical significance of these improvements.

A paired *t*-test was performed in the five cross-validation folds to evaluate the CPM differences between the proposed detector and competing state-of-the-art models. The proposed approach yielded significantly higher CPM values (*p* < 0.01), indicating a consistent improvement in detection sensitivity between nodules of varying sizes.

These findings demonstrate that the performance gains of Seg-CADe-CADx are statistically significant and are not attributable to random variation.

### 5.16. Inference and Deployment Feasibility

To evaluate deployment readiness, the full Seg-CADe-CADx pipeline was benchmarked on a standard workstation GPU. The average inference time was approximately 4 s per LDCT scan, with peak memory usage of about 3.2 GB. These values fall within the typical constraints of PACS-integrated CAD systems, which generally allow 10–15 s per scan. The model also supports a slice-wise inference mode that further reduces memory usage, and lighter variants of the model (e.g., without radiomics fusion) decrease parameter count with minimal impact on accuracy. These observations indicate that the framework is practical for real-time or batch-mode clinical deployment.

## 6. Discussion

The experimental findings demonstrate that the Seg-CADe-CADx framework effectively addresses persistent challenges in lung cancer screening by unifying segmentation-guided detection, hybrid CNN–Transformer classification, and probability calibration within a single pipeline. Although the segmentation module achieved a modest Dice coefficient of 0.742 and IoU of 0.591, these values were sufficient to generate anatomically meaningful candidate masks that constrained the search space and reduced false positives. This confirms that segmentation stability, rather than pixel-level precision, is the key requirement for downstream CADe performance.

For nodule detection, the framework achieved a CPM of 0.944 on LUNA16, with sensitivity of 91.0% for nodules ≤6 mm, 95.5% for 6–10 mm, and 98.9% for nodules >10 mm. High sensitivity for sub-6 mm nodules is clinically important because such lesions frequently correspond to early-stage malignancies that benefit most from early intervention. These results surpass those reported for SCPM-Net, multi-scale attention detectors, modified Faster R-CNN variants, and other recent hybrid detectors such as U-Net+YOLOv8+Swin and modified 3D-RPN+Transformer models. The improvement can be attributed to two factors: (1) the segmentation-guided proposal mechanism, which stabilizes small-nodule localization, and (2) the 2.5D refinement head, which captures inter-slice context while maintaining computational efficiency.

For malignancy classification, the DenseNet–Swin hybrid achieved ROC-AUC = 0.988, PR-AUC = 0.947, and accuracy of 93.4% on LIDC-IDRI. At the Youden threshold, sensitivity reached 96.6% with specificity of 92.9%. When operating at 95% sensitivity—clinically desirable for ruling out malignancy—specificity remained high at 93.8%. These results exceed those of recent CNN-, attention-, and Transformer-based CADx models, including MTST and GC-WIR, which either lack multi-scale integration or do not leverage segmentation-guided CADe context. Confusion-matrix analysis confirmed very few false negatives, while multitask prediction of radiological attributes (spiculation, lobulation) aligned the model’s internal reasoning with radiologist interpretation, enhancing clinical transparency.

Calibration results further validated the reliability of the malignancy scores, with a Brier score of 0.083 and ECE of 0.209. Reliability diagrams exhibited strong agreement between predicted probabilities and empirical frequencies, an essential property for clinical workflows where calibrated thresholds determine follow-up imaging, biopsy, or discharge decisions. The ablation analysis demonstrated that DenseNet excels at modeling local nodule texture (ROC-AUC = 0.972), while Swin captures long-range global structure (ROC-AUC = 0.981). Their hybrid integration delivered the highest discriminatory performance (ROC-AUC = 0.993). Although incorporating radiomics slightly reduced AUC to 0.988, it improved calibration smoothness and interpretability.

## 7. Conclusions

Seg-CADe-CADx introduces a unified framework that integrates segmentation-guided detection, a hybrid DenseNet–Swin Transformer classifier, and calibrated malignancy estimation for pulmonary nodule analysis. Evaluation on LUNA16 and LIDC-IDRI demonstrated high sensitivity for small nodules, superior classification accuracy (ROC-AUC = 0.988), and trustworthy calibrated outputs, all achieved with computational feasibility suitable for screening practice. By addressing both diagnostic accuracy and clinical interpretability, the framework provides a robust solution for LDCT-based lung cancer screening.

Future directions include extending validation to diverse multi-center cohorts, integrating longitudinal CT scans to capture temporal nodule evolution, and exploring federated learning for improved generalization without compromising patient privacy. Deployment-focused optimizations such as pruning and knowledge distillation will further enhance efficiency, supporting adoption in both advanced clinical centers and resource-limited environments.

## Figures and Tables

**Table 1:** Representative CADe results (2022–2025) with dataset diversity and external evaluation.

Year	Method	Dataset(s)	Metrics	Performance
2022	SCPM-Net (Anchor-free) [11]	LUNA16	CPM (0.125–8 FP/scan)	0.892
2022	Multi-scale Attention Detector [12]	LUNA16	CPM; Sensitivity	0.927; 94.5–96.2%
2023	Improved Faster R-CNN + OHEM [13]	LUNA16	Precision; Recall; F1	0.907; 0.568; 0.913
2024	Modified 3D-RPN + Transformer [14]	LUNA16 + NLST	CPM; Sens@8 FP/scan	0.901 (LUNA16); ∼0.72 (NLST)
2025	U-Net + YOLOv8 + Swin [15]	LUNA16 + Private	Precision; Recall; mAP@0.50	0.898; 0.851; 0.879
2025	Transformer-based CADe [16]	Tianchi	FROC; Sens@1 FP/scan	0.918; 93.4%
Proposed	Seg-CADe-CADx	LUNA16 + NLST	CPM; Sens@1 FP/scan	0.944; 96.4%

**Table 2:** Statistical comparison of the proposed method versus baseline models.

Model	Metric	Mean	95% CI	*p*-Value
Baseline CNN	AUC	0.954	[0.945, 0.962]	–
Swin CADx	AUC	0.972	[0.963, 0.979]	0.018
Seg-CADe-CADx (Proposed)	AUC	0.988	[0.983, 0.992]	<0.01
3D-RPN Detector	CPM	0.901	[0.883, 0.920]	–
YOLOv8 + Swin Detector	CPM	0.879	[0.861, 0.898]	0.044
Seg-CADe Detector (Proposed)	CPM	0.944	[0.931, 0.958]	<0.05

Notes: The symbol “–” denotes the reference baseline model against which statistical comparisons were performed; therefore, no *p*-value is reported for these entries.

**Table 3:** Datasets and evaluation protocols.

Dataset	CT Scans	Nodules	Annotations	Labels Used	Evaluation Metrics
LUNA16	888	>1000 (≥3 mm)	Consensus annotations (10-fold splits)	Centers, radii	FROC, CPM, size-stratified sensitivity
LIDC-IDRI	1018	7371 (≥3 mm)	Multi-radiologist, malignancy 1–5	Binarized malignancy (0 = benign, 1 = malignant)	ROC-AUC, PR-AUC, Acc, Sens/Spec, ECE

**Table 4:** Segmentation performance of the residual 3D U-Net (Stage 0).

Metric	Mean
Dice	0.742
IoU	0.591
HD95 (mm)	2.43

**Table 5:** Detection results on LUNA16 (overall sensitivity and CPM).

FP/Scan Range	Sensitivity	CPM
0.125–8	94.4%	0.944

**Table 6:** Size-stratified sensitivity at 1 FP/scan on LUNA16.

Nodule Size	Sensitivity	TP	FP	FN
≤6 mm	91.0%	457	296	45
6–10 mm	95.5%	385	364	18
>10 mm	98.9%	278	430	3

**Table 7:** Comparison of detection performance with prior methods on LUNA16.

Method	Dataset	CPM	Sens @1 FP/Scan	Notes
SCPM-Net (anchor-free, 2022) [11]	LUNA16	0.892	87.3%	Small nodule recall
Multi-scale attention (2022) [12]	LUNA16	0.927	94.5%	Attention boosted
Faster R-CNN + OHEM (2023) [13]	LUNA16	0.901	91.2%	Requires FPR head
U-Net + YOLOv8 + Swin (2025) [15]	LUNA16	0.879	85.1%	Hybrid but limited
Seg-CADe-CADx (Proposed)	LUNA16	**0.944**	**96.4%**	Best small-nodule sensitivity

Notes: Bold values indicate the best performance achieved for the corresponding evaluation metric.

**Table 8:** Comparison of CADx methods on LIDC-IDRI.

Method	Acc (%)	Sens (%)	Spec (%)	ROC-AUC	PR-AUC
NoduleX (CNN+Radiomics, 2018) [8]	92.0	91.3	90.2	0.990	0.982
3D-MCN Capsule (2020) [17]	93.1	94.9	90.0	0.964	0.951
MTST (Swin, 2024) [9]	93.7	91.6	96.1	0.982	0.974
GC-WIR (Attention, 2025) [18]	94.3	91.5	93.7	0.985	0.976
Seg-CADe-CADx (Proposed)	96.9	95.8	97.8	0.988	0.947

**Table 9:** Calibration comparison between models. Lower values indicate better calibration.

Model	ECE	Brier Score
Uncalibrated DenseNet–Swin	0.317	0.112
CNN-only classifier	0.284	0.101
Swin-only classifier	0.261	0.094
Seg-CADe-CADx (Calibrated)	0.209	0.083

**Table 10:** Ablation study results on LIDC-IDRI.

Configuration	ROC-AUC
DenseNet only	0.972
Swin only	0.981
DenseNet + Swin (no radiomics)	0.993
DenseNet + Swin + Radiomics	0.988

**Table 11:** Statistical significance results for comparative and ablation experiments. Values denote *p*-values from paired *t*-tests.

Comparison	ROC-AUC	Accuracy	CPM
Seg-CADe-CADx vs. Best CADx Baseline	<0.01	<0.05	<0.01
Seg-CADe-CADx vs. No-Radiomics Variant	0.02	<0.05	0.03
Seg-CADe-CADx vs. No-Segmentation Variant	<0.01	0.01	<0.01

**Table 12:** Comparison of model complexity and runtime.

Model	Params (M)	FLOPs (G)	Runtime (s/scan)
Faster R-CNN (2023)	32.1	120.5	4.2
Swin-only (2024)	28.4	110.2	3.8
Seg-CADe-CADx (Proposed)	36.5	124.8	4.0

**Table 13:** External validation results on an NLST subset. Seg-CADe-CADx was trained on LUNA16 and LIDC-IDRI and evaluated on NLST without fine-tuning.

Dataset	CPM (Detection)	ROC-AUC (Malignancy)	Sensitivity
LIDC-IDRI (Internal)	0.942	0.988	0.966
NLST (External)	0.903	0.951	0.884

## Data Availability

The data presented in this study are available from the corresponding author upon reasonable request.
